# Integrated Gut–Brain Axis Response to Freezing and Recovery in Freeze-Tolerant Fish, *Perccottus glenii*

**DOI:** 10.3390/ani16091338

**Published:** 2026-04-27

**Authors:** Ye Huang, Jiajun Zhou, Weichen Wang, Zhaoyang Ning, Xiangxin Kong, Kaitong Zhu, Zhitao Liu, Weijie Mu

**Affiliations:** Key Laboratory of Biodiversity of Aquatic Organisms, College of Life Science and Technology, Harbin Normal University, Harbin 150025, China; fieldhwang@163.com (Y.H.); 16603097309@163.com (J.Z.); wangweichen2021@126.com (W.W.); 13275358746@163.com (Z.N.); kongxx2021@126.com (X.K.); zkt757903@163.com (K.Z.); zzhaol2021@126.com (Z.L.)

**Keywords:** *Perccottus glenii*, brain–gut axis, recovery from freezing, transcriptomic, gut microbiota

## Abstract

The molecular mechanisms underlying freezing recovery in vertebrates, particularly in teleost fish, remain inadequately understood. This study utilized an integrated approach combining brain transcriptomics and gut microbiota analysis to investigate the recovery process in the freeze-tolerant species *Perccottus glenii* following experimental freezing and thawing. Significant transcriptomic reprogramming was observed in brain tissue, with the PPAR signaling pathway strongly activated immediately upon thawing (R0), potentially facilitating lipid metabolism and neuroprotection during ischemic stress. After 4 h of recovery (R4), circadian rhythm pathways were significantly upregulated, suggesting a potential role in coordinating metabolic recovery during reperfusion. Concurrently, gut microbiota analysis revealed substantial community shifts, characterized by a marked decrease in Bacillota and an increase in Pseudomonadota during the initial recovery phase. These findings suggest that successful freezing recovery in *P. glenii* is associated with coordinated changes in brain and gut, highlighting potential roles of metabolic adaptation and microbial ecological dynamics under the specific freezing protocol employed.

## Simple Summary

The ability of freeze-tolerant fish to survive freezing and recover afterwards is not fully understood at the molecular level. In this study, we aimed to investigate the changes occurring in the brain and gut bacteria of the fish *Perccottus glenii* during the first hours of recovery from freezing. We found that immediately after warming begins, the fish’s brain activates a specific pathway involved in fat metabolism. This activation likely helps protect nerve cells from damage caused by a lack of oxygen. After four hours of recovery, the brain shifts toward regulating daily biological rhythms. This shift suggests a role in coordinating overall metabolic restoration. At the same time, the community of bacteria in the gut undergoes major shifts. Some common bacterial groups decrease while others increase. These results indicate that successful recovery from freezing involves coordinated adjustments in both the brain and the gut microbes. Understanding these natural mechanisms is valuable to society because it may inspire new methods for preserving human organs for transplantation or for improving the storage of biological materials. Such knowledge could ultimately help reduce cell damage and save lives.

## 1. Introduction

Cold resistance primarily occurs in high-latitude regions, enabling local species to endure prolonged freezing stress [1]. Among these adaptations, freeze tolerance allows biological tissues to regain vitality and recover after freezing and thawing, while freeze avoidance prevents freezing through various physiological and biochemical mechanisms. These adaptations provide protection at relatively low temperatures [2]. The forest frog (*Rana sylvatica*) can survive even in a completely frozen state. Once the environmental temperature rises, the forest frog can autonomously thaw within just a few hours, fully restoring its physiological functions and reviving vigorously [3]. *Antarctic notothenioids*, which evolved in the extremely cold environment of Antarctica, represent another group of cold-resistant species. They are rich in antifreeze proteins (AFPs) and antifreeze protectants, which effectively inhibit ice crystal growth and mitigate ice crystal formation, thereby maintaining cellular structural integrity [4,5]. Amphibians that live in frigid habitats with temperatures below freezing have consistently drawn considerable interest from researchers because of their extraordinary capacity to endure and withstand freezing conditions [1,6,7]. The key to this adaptability lies in their unique physiological mechanisms, which enable them to maintain basic functions at low temperatures and successfully resist freezing events [8]. Critically, the recovery process from this frozen state parallels ischemia–reperfusion events: hibernation resembles ischemia, while thawing and reperfusion involve similar physiological stresses [9,10,11,12,13]. Ischemia–reperfusion has been implicated in processes such as blood–brain barrier disruption [14]. In contrast to amphibians, research on the molecular regulatory mechanisms of fish during recovery from freezing remains relatively scarce. Analyzing how fish successfully recover after experiencing tissue freezing can illuminate survival strategies and adaptive limits in extreme environments.

The Chinese sleeper perch (*Perccottus glenii*) is a freeze-tolerant teleost fish capable of surviving complete encasement in ice during winter [15], representing one of the few vertebrates outside of Reptilia and Amphibia with this remarkable ability. In addition, this species is native to Northeast Asia and prevalent in the Heilongjiang River of China [16,17]. *P. glenii* possesses tender, flavorful, boneless meat with high nutritional value, making it highly marketable; however, the lack of basic research critically impedes artificial breeding efforts [18]. *P. glenii* exhibits tolerance to extreme stressors, including hypoxia and complete freezing [19,20]. During freezing, vital physiological functions cease; however, individuals can fully recover within hours post-thaw [21]. Crucially, this process of freezing and thawing parallels the pathological states of ischemia and reperfusion, posing a dual challenge of energy depletion and oxidative stress. This exceptional tolerance to freezing and recovery makes *P. glenii* an ideal model for investigating the molecular mechanisms underlying recovery [17,20]. Consequently, it is particularly suitable for exploring the role of the brain–gut axis in coordinating physiological recovery following freezing stress.

The intricate symbiotic relationship between animal hosts and their gut microbiota, conceptualized as “holobionts” [22], significantly influences host physiology, evolution, and adaptation [23,24]. This dynamic association, rather than being merely coincidental, represents a finely tuned collaboration shaped by evolutionary pressures, impacting critical functions including digestion, energy metabolism, immune regulation, and disease susceptibility [25,26,27,28]. Exogenous factors such as diet, pharmaceuticals, and environmental pollutants profoundly modulate the gut microbiota [29,30]. While research on homeothermic animals is relatively advanced, significant knowledge gaps persist regarding the gut microbiota of poikilothermic (“cold-blooded”) vertebrates (such as amphibians, fish, and reptiles) and invertebrates, particularly concerning their responses to environmental stressors [31]. However, the aforementioned evidence is primarily concentrated in amphibians and insects, and there remains a lack of in-depth research on whether and how the gut microbiota of freeze-tolerant fish, which similarly face freezing stress, participate in host cold adaptation and recovery. This is especially pertinent during physiologically demanding states like hibernation or brumation, where prolonged low temperatures, fasting, and depressed metabolic rates drastically alter host physiology and likely reshape microbial communities [32]. The gut microbiota is hypothesized to play crucial roles in meeting energy demands and facilitating adaptation to fluctuating energy availability throughout these periods and during subsequent arousal [32]. Furthermore, sustained shifts in the gut microbiota during hibernation may trigger complex physiological adaptations that impact multiple life stages. Although temperature increases are known to affect gut microbiota diversity and stability in various animals [33,34,35,36], the impact of sustained low or extreme cold temperatures on these communities remains poorly understood. Understanding microbial responses to extreme cold in poikilotherms is critical, as gut microbiota may confer essential protective functions for host survival. The quantity of bacteria belonging to the Lachnospiraceae family markedly rose after being subjected to freezing temperatures, presumably aiding the survival of *Nanorana parkeri* in cold conditions [10]. Similarly, specific microbes with ice-nucleating activity, such as *Fusarium* sp. found in moth larvae [37,38], and ice-nucleating bacteria in overwintering *Rana sylvatica* [38], are known to facilitate controlled extracellular freezing at higher sub-zero temperatures, thereby enhancing freeze tolerance. However, existing findings primarily focus on individual taxa or functions. A comprehensive understanding of how overall microbial diversity, community structure, and functional potential adapt to support poikilothermic hosts under extreme cold stress is still lacking. This adaptation is particularly crucial for meeting the heightened energy demands and mitigating the oxidative damage associated with ischemia–reperfusion during hibernation and arousal. The gut and the brain are interconnected through neural and humoral systems, forming a complex bidirectional communication system known as the “gut–brain axis” [39,40]. Recent studies have demonstrated that temperature stress can impact both intestinal and brain functions, while the host can regulate heat production capacity and cold adaptation via the gut–brain axis [41]. Given that the gut and the brain are interconnected through neural and humoral systems, forming a complex bidirectional communication system known as the “gut–brain axis,” we hypothesize that the gut microbiota plays a critical role in modulating the host’s physiological recovery from freezing stress. Specifically, we propose that changes in microbial communities during the ischemic (frozen) state may support the brain’s metabolic and neuroprotective responses during reperfusion (thawing) via this axis, potentially through modulating the availability of energy substrates or producing signaling molecules. To test this hypothesis, this study investigates the temporal dynamics of both the brain transcriptome and gut microbiota in *P. glenii* during recovery from freezing, aiming to potentially identify coordinated and potentially causal interactions within the gut–brain axis.

This study investigates *P. glenii* as the research subject, focusing on recovery from freezing experiments. Recovery from the freezing process was conducted at 4 °C, with samples collected at three time points: control group (CK), recovery from freezing at 0 h (R0), and recovery from freezing at 4 h (R4). A comparative transcriptome analysis was performed using intestinal and brain samples from *P. glenii* at these time points, followed by enrichment analyses of differentially expressed genes (DEGs), Gene Ontology (GO), and Kyoto Encyclopedia of Genes and Genomes (KEGG). Gene set enrichment analysis was employed to explore the differential regulatory patterns in the brain axes of *P. glenii* under recovery from freezing conditions at CK, R0, and R4. The present study integrates brain transcriptome and gut microbiota temporal dynamics to investigate the gut–brain axis during recovery from freezing in *P. glenii*, specifically revealing the coordinated molecular and microbial responses that underlie the transition from frozen to recovery states for the first time. This study enhances our understanding of the molecular response mechanisms involved in the recovery from freezing of *P. glenii*, providing a theoretical foundation for the physiological regulatory mechanisms of cold-tolerant fish.

## 2. Materials and Methods

### 2.1. Fish, Recovery from Freezing Challenge

**Figure 1:**
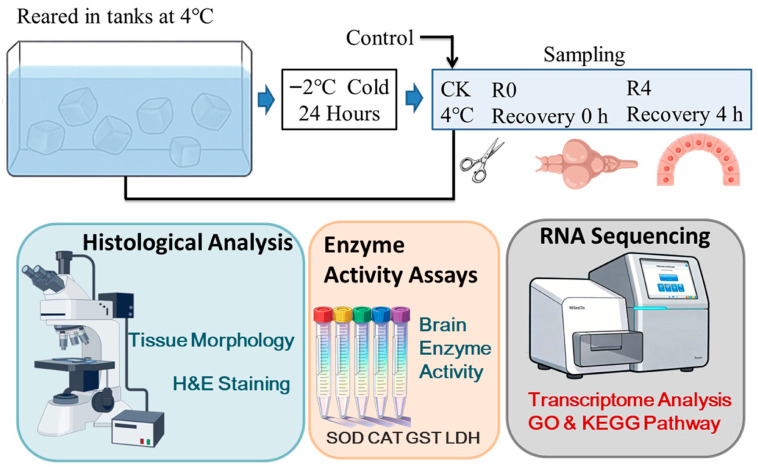
Experimental design.

The Amur sleeper (*Perccottus glenii*) was obtained from Harbin Normal University. The individuals involved in the experiments had an average body weight of 32.7 ± 4.12 g. A total of 108 fish were used in this experiment. Three treatment groups were established: control (CK, without freezing at 4 °C), 0 h post-freezing (R0), and 4 h post-freezing (R4). Each treatment group consisted of three replicate tanks, with 12 fish per tank, yielding 36 fish per group. The fish were raised in tanks, each with a capacity exceeding 90 L of usable water maintained at 4 °C. The process for recovering *P. glenii* after freezing was conducted following the established protocols from our previous studies [42,43]. The fish were fed earthworms to satiation, with the cooling rate set at 1 °C/h. After reaching −2 °C, the cooling was stopped, and the samples were maintained at this temperature for 24 h. Water temperature was strictly maintained at 4 °C for the control group following our previous study [42]. After freezing, the fish were transferred to 4 °C water for recovery, and samples were collected at 0 h and 4 h post-recovery. Sampling was then conducted at control (without freezing at 4 °C, CK), 0 h (R0), and 4 h (R4), as shown in the experiment design in Figure 1. From each group (36 fish), six randomly selected fish were used for histological analysis of brain and gut tissues (*n* = 6). Another six randomly selected fish were used for gut microbiota analysis (*n* = 6), for which intestinal contents were collected fresh. Six additional randomly selected fish from each group were used exclusively for brain enzyme activity assays (*n* = 6). Finally, three randomly selected fish from each group were used exclusively for brain transcriptome sequencing (*n* = 3). All samples were collected from individual fish without pooling across individuals. Before the sampling, the fish were sedated using MS-222 at a concentration of 200 mg/L. All experimental methods complied with institutional guidelines for the ethical treatment of laboratory animals. Subsequently, each fish was carefully dissected using sterilized scissors on a sterile ice board, and the brain, gut, and intestinal contents were collected for further examination. All animal research protocols received prior approval from the Harbin Normal University Animal Care Committee (HNUARIA2024024).

### 2.2. Histological Analysis of the Brain and Gut Samples

The brain and gut tissue samples were fixed in Bouin’s fixative and then placed in ethanol for dehydration. The fixed tissues were sequentially embedded in paraffin. Paraffin blocks were adjusted to appropriate dimensions, and thin sections were sliced to a thickness of 5 μm utilizing a microtome. These sections were subsequently immersed in a water bath at 40 °C to facilitate stretching and were then allowed to dry overnight at 38 °C. After the application of hematoxylin and eosin (H&E) stains, the sections were mounted with neutral balsam and left to dry overnight in a fume hood. Observations and photographs were captured under a microscope. For each fish, three non-consecutive sections from the brain and three from the midgut were analyzed to account for technical replicates. The observers conducting the morphological measurements were blinded to the experimental group identities. ImageJ software v. 2.1.0/1.53c (https://imagej.net/Fiji/Downloads, accessed on 2 November 2024) was used for morphometric analysis; prior to measurements, the software was calibrated using a stage micrometer image captured under the same magnification, and the scale was set uniformly for all images. Intestinal and brain tissue morphology was also evaluated across these experimental conditions. Following fixation, samples underwent sequential ethanol dehydration, xylene clearing, and paraffin embedding. Essential morphometric metrics, including the brain’s periventricular stratum (SPV), central white matter layer (SAC), central gray matter layer (SGC), superficial fibrous and gray layer (SFGS), optic layer (SO), marginal layer (SM), intestinal fold height (IFH), mucosal thickness (MT), and thickness of the muscular layer (MLT), were derived from analyses conducted using ImageJ software (https://imagej.net/Fiji/Downloads, accessed on 2 November 2024). This approach ensures that tissue heterogeneity is adequately sampled and reduces potential bias in data selection.

### 2.3. Determination of Brain Enzyme Activity

Brain tissue samples were homogenized in pre-chilled physiological saline (0.9% NaCl) on ice at a tissue-to-solution ratio of 1:9 (*w*/*v*). Homogenization was performed using a glass homogenizer maintained in an ice–water bath until the tissue was thoroughly disrupted. The resulting slurry was transferred to centrifuge tubes and centrifuged at 10,000× *g* for 10 min at 4 °C. The supernatant was then collected into EP tubes for subsequent enzyme activity assays. SOD activity was measured using the nitroblue tetrazolium (NBT) reduction method at 560 nm. CAT activity was determined based on the formation of a stable complex with ammonium molybdate, monitored at 405 nm. GPx activity was measured by monitoring NADPH oxidation at 340 nm using a coupled assay with GR (glutathione reductase), GSH (reduced glutathione), and 2.5 mM H_2_O_2_ in potassium phosphate (KPi) buffer (pH 7.0), with blanks omitting the supernatant. LDH activity was measured at 340 nm and 25 °C in a reaction mixture containing 0.1 M phosphate buffer (pH 7.5), NADH (2 mg/mL), supernatant, and sodium pyruvate. Enzyme activities were expressed in units per milligram of protein per minute (U·mg protein^−1^·min^−1^), where one unit (U) is defined as a change of 0.01 in optical density per minute.

### 2.4. RNA Extraction, Sequencing, and Annotation of Brain

The raw data were submitted to the NCBI Sequence Read Archive (SRA), which is identified by BioProject accession number PRJNA1330386. We extracted total RNA from each brain sample with TRIzol^®^ reagent (Invitrogen, Carlsbad, CA, USA) and measured the concentration using both agarose gel electrophoresis and a NanoDrop2000 spectrophotometer (Thermo Scientific, Waltham, MA, USA). For cDNA synthesis, we utilized the TransScript^®^ All-in-One First-Strand cDNA Synthesis SuperMix for qPCR (One-Step gDNA Removal) (TransGen Biotech, Beijing, China). To remove rRNA, we applied the Ribo-Zero rRNA removal kit (Epicentre, Madison, WI, USA). cDNA libraries were sequenced by Genedenovo Biotechnology Co., Ltd. (Guangzhou, China) on an Illumina platform. Subsequently, we filtered the raw reads using FASTP (Version 0.18.0) to yield high-quality clean reads by eliminating those containing adapters. In addition, reads containing more than 50% low-quality bases and reads containing more than 10% unknown nucleotides (N) were excluded. After filtering, the reads were mapped to the reference genome of *P. glenii* (NCBI Genome Database ID10710, GCA_024416835.2) using HISAT 2.4 version. Gene expression levels were determined utilizing the TPM (Transcripts Per Kilobase of exon model per Million mapped reads) approach. Differentially expressed genes (DEGs) were identified using the DESeq2 version 1.42.0 tool, applying a significance threshold of *p* < 0.05 and a fold change criterion (|log2FC| ≥ 1) [44]. To identify significant enrichment among DEGs, both Gene Ontology (GO) functional enrichment and Kyoto Encyclopedia of Genes and Genomes (KEGG) pathway analyses were executed, again using *p* < 0.05 as the criterion for significance. Specifically, KEGG pathway analysis was performed with reference to the KEGG database, and GO functional enrichment analysis was conducted using the GO database.

### 2.5. Quantitative PCR (qPCR) Validation

A total of seven differentially expressed genes (DEGs) were randomly selected, and their relative expression levels were quantified by qPCR to verify the results obtained from the transcriptome analysis. Brain tissue RNA from the fish was extracted using the TRIzol method. Agarose gel electrophoresis and an ultramicro nucleic acid detector were used to assess RNA integrity and concentration, respectively. Subsequently, cDNA was synthesized using a one-step cDNA synthesis kit (TransGen Biotech, Beijing, China). RT-qPCR was subsequently executed using SYBR Green Supermix (TransGen, Beijing, China) on a LightCycler^®^96 real-time PCR system (Roche, Basel, Switzerland). The primers employed in the qPCR analysis, along with their amplification efficiencies, are detailed in Table 1. The relative expression levels of each gene across various tissues were determined using the 2^−ΔΔCt^ method, normalizing against the endogenous control gene β-actin, known for its relatively stable expression in *P. glenii* [17].

### 2.6. Microbial Analysis of the Gut Samples

Upon sampling, the gut contents were carefully collected and washed with PBS solution. The extraction of microbial genomic DNA was conducted using the MOBIO PowerSoil^®^ DNA Isolation Kit (MoBio Laboratories, Carlsbad, CA, USA). The hypervariable V3-V4 regions of the 16S rDNA gene were amplified from the extracted genomic DNA to construct libraries. Subsequently, the total PCR products were quantified with Quant-iT™ dsDNA HS reagent (Thermo Fisher Scientific Inc., Waltham, MA, USA). To enable a comprehensive analysis of bacterial rRNA genes, high-throughput sequencing (2 × 250 paired-end) was executed on the Illumina MiSeq platform. Sequence data analysis was carried out using QIIME2 (version 2023.9), enhanced by the R package (version 3.2.0). For evaluating α-diversity, indices such as Chao1, Shannon, and Simpson were calculated. The structural variations in the microbial communities were examined through Bray–Curtis-based β-diversity analysis. Visualization methods, including principal coordinate analysis (PCoA), non-metric multidimensional scaling (NMDS), and unweighted pair group method with arithmetic mean clustering, were employed. Phylogenetic understanding of the communities was derived using PICRUSt2, which entailed standardizing the OTU table to reduce bias arising from variations in 16S rRNA gene copy numbers across species. Linear Discriminant Analysis Effect Size (LEfSe) was applied to detect biomarkers in the intestinal microbiota of *P. glenii*, with the cut-off values set at |LDA score| > 4 and *p* < 0.05.

### 2.7. Statistical Analysis

All experimental values in this study were expressed as mean ± standard deviation (M ± SD) (*n* = 6). Statistical analysis was conducted using GraphPad Prism software (version 10.6.1). The activities of SOD, CAT, GPx, and LDH were tested for normality using the Shapiro–Wilk test and for homoscedasticity using the Brown-Forsythe test. Statistical significance was analyzed using one-way ANOVA, followed by Tukey’s post hoc test. Statistical significance analysis was performed using one-way ANOVA analysis of variance, followed by Tukey’s test. Significant differences were set at *p* < 0.05 (*).

## 3. Results

### 3.1. Histological Results

**Figure 2:**
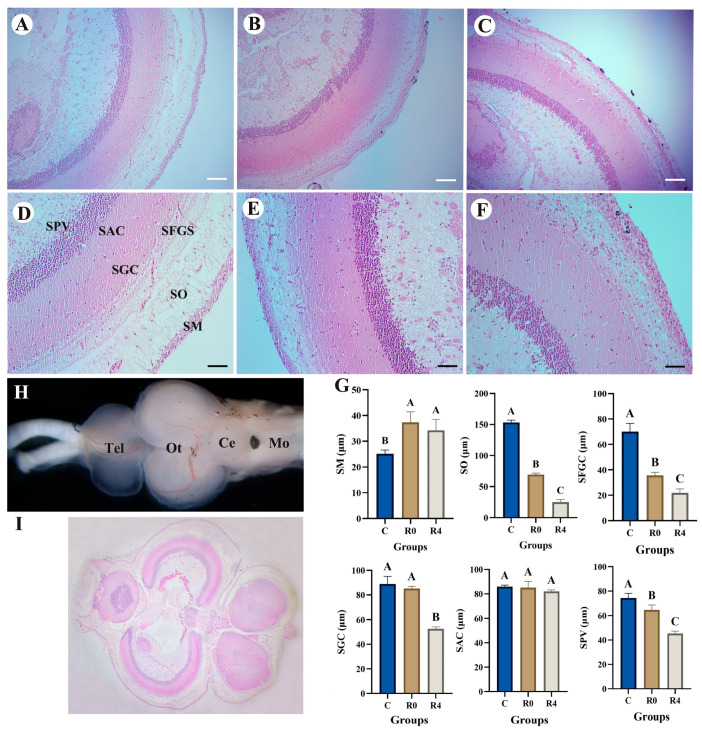
Histological structure of the fish brain in the experimental groups. (**A**,**D**) Control group. (**B**,**E**) Recovery from freezing at 0 h. (**C**,**F**) Recovery from freezing at 4 h. (**G**) The changes in the six-layer structure of the top cover in each group. (**H**) Schematic diagram of the morphology of the whole brain of *P. glenii*. (**I**) Schematic diagram of the H&E of the whole brain of *P. glenii*. SPV, stratum periventriculae; SAC, stratum album central; SGC, stratum griseum central; SFGS, stratum fibrosum et griseum superficiale; SO, stratum opticum; SM, stratum marginale. Vertical bars represented the mean ± SEM (*n* = 6). Data marked with letters differed significantly (*p* < 0.05) among groups. White bars = 40 μm. Black bars = 20 μm.

**Figure 3:**
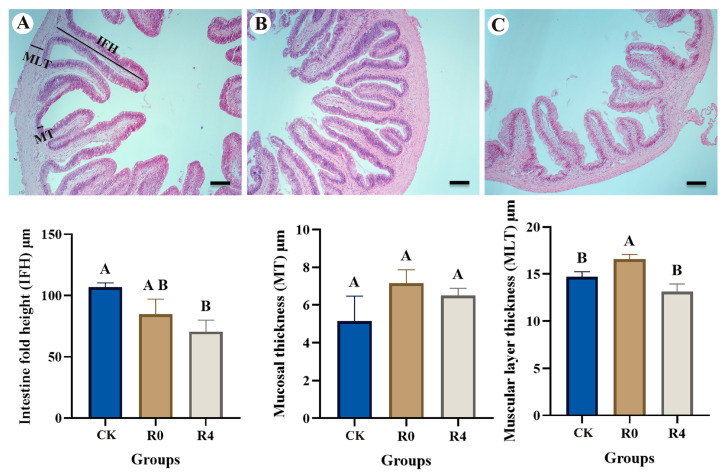
Histological structure of the fish intestine in the experimental groups. (**A**) Control group. (**B**) Recovery from freezing at 0 h. (**C**) Recovery from freezing at 4 h. Intestine fold height (IFH), mucosal thickness (MT), and muscular layer thickness (MLT) were determined. Vertical bars represented the mean ± SEM (*n* = 6). Data marked with letters differed significantly (*p* < 0.05) among groups. Bars = 40 μm.

The optic tectum exhibits the characteristic six-layer structure of bony fish (SM, SO, SFGS, SGC, SAC, SPV) (Figure 2). Notable variations in the thickness of each layer were observed among the experimental groups. Morphometric analysis was performed on the brain tissue structure of the control group (CK), the R0 and R4 groups, specifically examining the periventricular SPV, central white matter SAC, central gray matter SGC, superficial fibrous gray matter SFGS, visual layer SO, and marginal layer SM after freezing at 4 °C. The results indicated that, compared to the control group (CK), there were no significant differences in SGC and SAC between the R0 group and the control group, while the thicknesses of SO, SFGS, and SPV were significantly reduced (*p* < 0.05, Figure 2). Conversely, at 4 h of recovery (Group R4), the thickness of SM was significantly greater than that of Group CK (*p* < 0.05, Figure 2), whereas SO, SFGS, and SPV were significantly lower than those in both Group CK and Group R0 (*p* < 0.05, Figure 2), and SGC was significantly reduced compared to Group CK (*p* < 0.05, Figure 2). These findings suggest that, after four hours of recovery from freezing, the thickness of most brain tissue layers significantly decreased (*p* < 0.05, Figure 2), with the exception of the SAC layer, which showed no significant change (*p* > 0.05). Furthermore, significant differences in intestinal tissue morphological indicators of *P. glenii* were observed among the different treatment groups (CK, R0, and R4) (*p* < 0.05, Figure 3). Specifically, IFH was significantly higher in the control group compared with the R4 group (*p* < 0.05, Figure 3), while no significant differences were found between the R0 group and either the CK or R4 group (*p* > 0.05, Figure 3). There was no significant difference in MT among the three groups (*p* > 0.05, Figure 3). However, MLT was significantly higher in group R0 compared to both group CK and group R4 (*p* < 0.05, Figure 3). In contrast, no significant difference in MLT was observed between group CK and group R4 (*p* > 0.05, Figure 3).

### 3.2. Enzyme Activity

**Figure 4:**
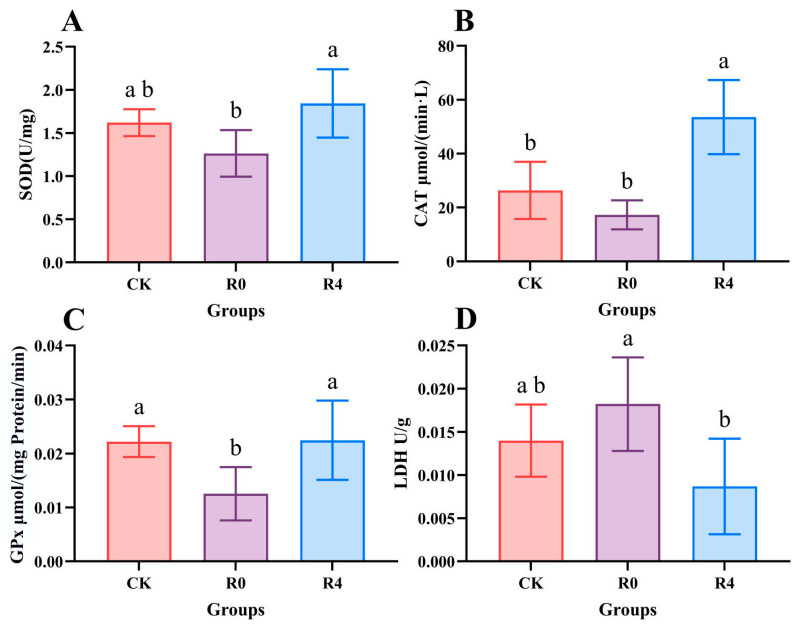
Brain antioxidant-related enzyme activities during recovery from freezing in the *P. glenii*. All data are presented as mean ± standard error (*n* = 6). Significant differences among treatments are indicated by different superscript letters (*p* < 0.05). (**A**) SOD activity (**B**) CAT activity (**C**) GPx activity (**D**) LDH activity.

The SOD activity in both the CK and R4 groups was significantly higher than that in the R0 group (Figure 4, *p* < 0.05). However, no significant difference in SOD activity was observed between the CK and R4 groups (*p* > 0.05). Additionally, CAT activity in the R4 group was significantly elevated compared to both the CK and R0 groups (*p* < 0.05), while that in the CK group was also significantly higher than that in the R0 group (*p* < 0.05). Furthermore, GPx activity in both the R4 and CK groups was significantly greater than that in the R0 group (*p* < 0.05), with no significant difference noted between the CK and R4 groups (*p* > 0.05). In contrast, LDH activity in the R0 group was significantly higher than that in both the CK and R4 groups (*p* < 0.05), with no significant difference in LDH activity observed between the CK and R4 groups (*p* > 0.05).

### 3.3. Transcriptome in the Brain of P. glenii

#### 3.3.1. Sequencing and Sequence Assembly Results

The average number of raw reads obtained from transcriptome sequencing for the CK, R0, and R4 groups was 40,106,711, 44,227,715, and 42,097,789, respectively, as shown in Table 2. Raw reads were processed to generate high-quality clean reads, with average final reads of 39,764,693, 44,177,323, and 41,730,599 for the CK, R0, and R4 groups, respectively. The GC content for each sample ranged from 43.19% to 47.09%. The Q20 and Q30 values, representing the proportions of bases with quality scores ≥20 and ≥30, respectively, ranged from 96.54% to 99.05% (mean: 97.50%) and from 90.46% to 97.37% (mean: 93.22%, Table 2).

#### 3.3.2. Transcriptome Annotation Analysis Results

**Figure 5:**
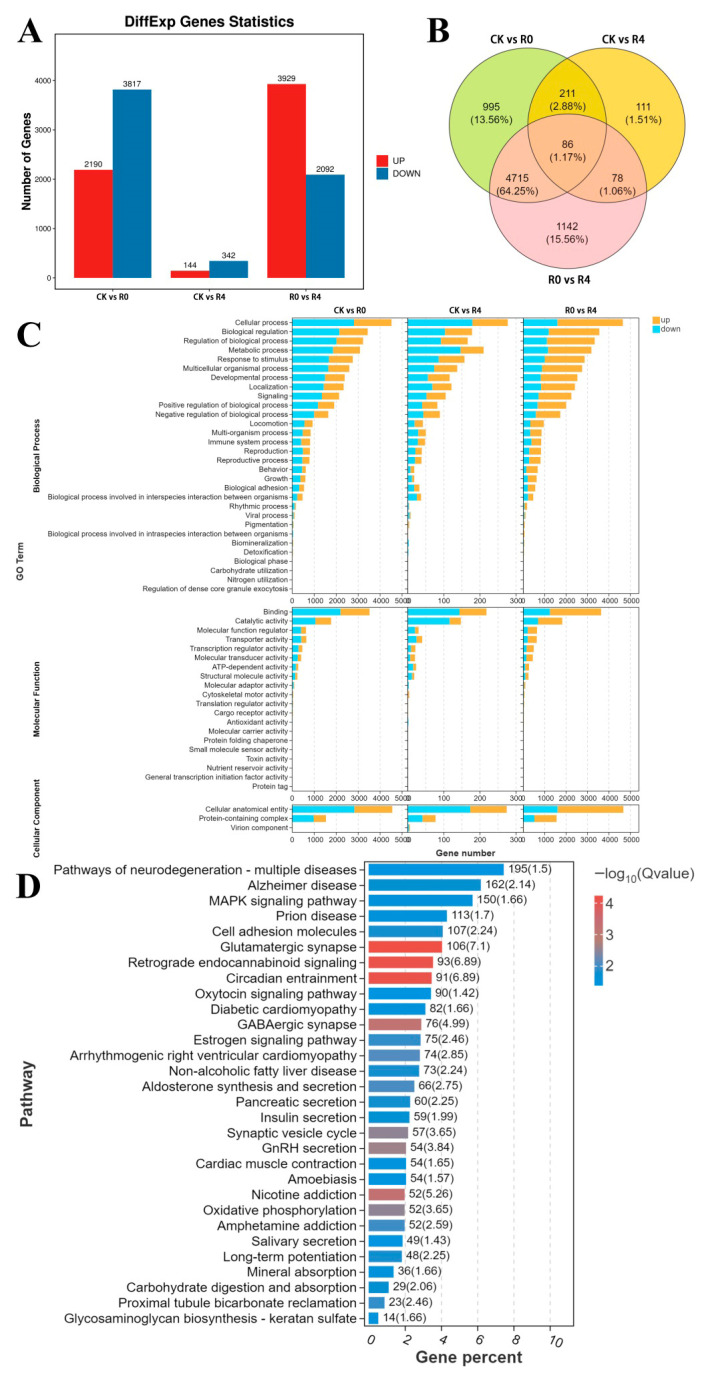
Transcriptome analysis of each comparison group. CK vs. R0 group, CK vs. R4 group, R0 vs. R4 group. (**A**) The number of DEGs in each comparison group. (**B**) Venn analysis of DEGs of each comparison group. (**C**) GO enrichment analysis of the DEGs in the brain transcriptome of the *P. glenii* (**D**).

Figure 3 shows 6007 DEGs between the CK and R0 groups, consisting of 2190 upregulated and 3817 downregulated genes (Figure 5A). In total, 486 DEGs were identified between the CK and R4 groups, with 144 upregulated and 342 downregulated genes. In addition, 6021 DEGs were identified between the R0 and R4 groups, with 3929 upregulated and 2092 downregulated genes. Furthermore, 86 shared DEGs were identified among the CK, R0, and R4 groups (Figure 5B). Gene Ontology function annotation results of DEGs in the brain transcriptome across three experimental cohorts (Figure 5C) revealed functional enrichment patterns associated with “Cellular process” and “Biological process”, which occupied a prominent place in the biological process category. At the level of molecular function, “Binding” and “Catalytic activity” were the most abundant enrichment terms. In terms of cellular components, the richest GO terms were “Cellular anatomical entity”. Kyoto Encyclopedia of Genes and Genomes (KEGG) enrichment analysis revealed the key pathways enriched by differentially expressed genes in different treatment groups, including the MAPK signaling pathway, cell adhesion molecules, glutamatergic synapse, retrograde endocannabinoid signaling, circadian entrainment, and diseases (Figure 5D).

#### 3.3.3. Analysis of Up- and Down-Regulated DEGs in the Brain of *P. glenii* During Recovery

**Figure 6:**
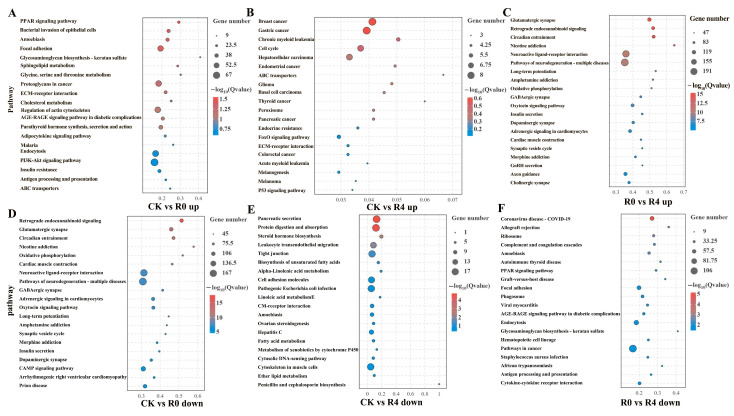
Kyoto Encyclopedia of Genes and Genomes (KEGG) enrichment analyses of differentially expressed genes in CK vs. R0, CK vs. R4, and R0 vs. R4. The size of the bubble indicates the number of differentially expressed genes in the pathway, and the color of the bubble changes from red to blue, indicating that the smaller the *p*-value of enrichment, the greater the significance. (**A**) CK vs R0 Up (**B**) CK vs R4 Up (**C**) R0 vs R4 Up (**D**) CK vs R0 Down (**E**) CK vs R4 Down (**F**) R0 vs R4 Down.

In the comparison between the R0 and CK groups, the pathway related to the “PPAR signaling pathway” was significantly enriched through the up-regulated genes (Figure 6A). In the comparison between the R4 and CK groups, pathways associated with “Breast cancer” and ‘’Gastric cancer” were significantly enriched through the upregulated genes (Figure 6B). In the R0 vs. R4 comparison, pathways related to metabolic processes, including “Glutamatergic synapse”, “Retrograde endocannabinoid signaling”, and “Circadian entrainment”, were significantly enriched through the upregulated genes (Figure 6C). In the R0 (ischemia) versus CK group, pathways related to “Retrograde endocannabinoid signaling” were significantly enriched through the downregulated genes (Figure 6D). In the comparison between the R4 and CK groups, pathways associated with “Pancreatic secretion” and “Protein digestion and absorption” were significantly enriched through the downregulated genes (Figure 6E). In the comparison between the R0 and R4 groups, pathways related to “Coronavirus disease—COVID-19” were significantly enriched through the down-regulated genes (Figure 6F). It should be noted that these KEGG pathway names refer to evolutionarily conserved signaling modules, such as breast cancer, gastric cancer, and Coronavirus disease (COVID-19), rather than to the human diseases themselves. As shown in Table S1, in the R4 group compared with the R0 group, circadian rhythm-related genes (PER1, PER2, PER3, RASD1, and MTNR1AA) were significantly upregulated. For melatonin signaling-related genes, MTNR1AA (melatonin receptor type 1A) was upregulated in both the CK vs. R4 and R0 vs. R4 comparisons, indicating a time-dependent and group-specific activation of melatonin receptor signaling (Table S1). Regarding lipid metabolism-related genes, PPARA, LPL, PCK2, EHHADH, SLC27A2, RXRBA, and RXRAA showed consistent upregulation in the CK vs. R0 comparison group (Table S1).

### 3.4. Validation of Gene Transcript Profiles by qRT-PCR

**Figure 7:**
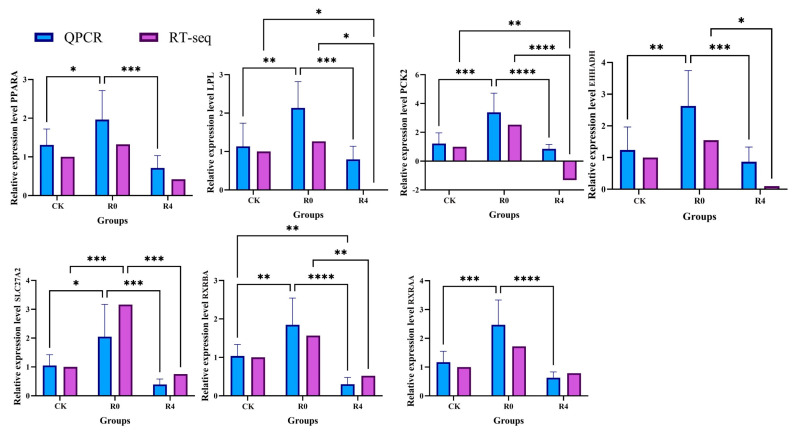
qRT-PCR analysis of seven genes for the validation of RNA-Seq data. **** *p* < 0.0001, *** *p* < 0.001, ** *p* < 0.01, * *p* < 0.05.

Seven differentially expressed genes (DEGs) were selected for validation through quantitative reverse transcription polymerase chain reaction (qRT-PCR) analysis, focusing specifically on genes associated with lipid metabolism (Figure 7). The transcript levels of PPARA, LPL, PCK2, EHHADH, SLC27A2, RXRBA, and RXRA exhibited significant differences among the treated groups, as demonstrated by both qRT-PCR and RNA sequencing (RNA-Seq) analyses. The results from the RT-qPCR analyses were highly consistent with those derived from the RNA-Seq data, thereby confirming the reliability of the sequencing results.

### 3.5. Analysis of Gut Microbiota Diversity in P. glenii

**Figure 8:**
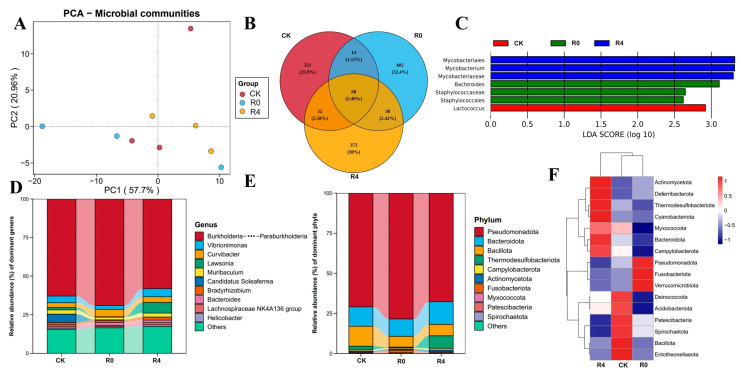
Gut microbiota in *P. glenii* in recovery from freezing. (**A**) Differences in the community composition of the three microbial sub-communities. Principal coordinates analysis (PCoA) based on the Bray–Curtis dissimilarity. (**B**) Venn diagram analysis at the ASV level of *P. glenii* gut microbiota in recovery from freezing. (**C**) LEfSe analysis of the intestinal microbiota. (**D**) Relative abundances of dominant bacteria in the intestines of *P. glenii* gut microbiota in recovery from freezing at the genus level. (**E**) Relative abundances of dominant bacteria in the intestines of *P. glenii* gut microbiota in recovery from freezing at the phylum level. (**F**) Heatmap of dominant bacteria in the intestines of *P. glenii* gut microbiota in recovery from freezing at the phylum level.

Principal Coordinates Analysis (PCoA) based on Bray–Curtis distances was employed to assess gut microbiota differences in *P. glenii* across various recovery time points from freezing (Figure 8A). The 16S rRNA amplicon sequences from the gut samples clustered into 1207 operational taxonomic units (OTUs), with 321 OTUs specific to the control group, 402 OTUs unique to the R0 group, 372 OTUs exclusive to the R4 group, and 68 OTUs shared among the three groups (Figure 8B). Utilizing thresholds of *p* < 0.05 and LDA > 4 from LEfSe, the key differential genera identified included *Lactococcus* in the CK group, *Bacteroides*, Staphylococcaceae, and Staphylococcales in the R0 group, and Mycobacteriales, *Mycobacterium*, and Mycobacteriaceae in the R4 group (Figure 8C). At the genus level, *Burkholderia-Caballeronia-Paraburkholderia* and *Vibrionimonas* were predominant across all groups. Furthermore, compared to the CK and R4 groups, the relative abundance of *Burkholderia-Caballeronia-Paraburkholderia* was significantly decreased in the R0 group. In addition, the relative abundance of *Vibrionimonas* was highest in the R4 group, followed by the CK group, and lowest in the R0 group (Figure 8D). At the phylum level, Pseudomonadota and Bacteroidota were prevalent in all groups. The relative abundance of Pseudomonadota was significantly higher in the R0 group compared to the CK group, but lower in the R4 group (Figure 8E). Heatmap analysis further indicated that Pseudomonadota was relatively less abundant in the CK group compared to the R0 group, while Actinomycetota was enriched in the R4 group (Figure 8F). Intriguingly, the peak relative abundance of Pseudomonadota in the R0 group coincided with the significant upregulation of the PPAR signaling pathway in the brain (Figure 6A), suggesting a potential temporal and functional correlation between shifts in gut microbial community composition and the host’s central energy metabolism during the R0 (ischemic) phase. This correlation supports the hypothesis of gut–brain involvement in the freezing recovery process.

## 4. Discussion

**Figure 9:**
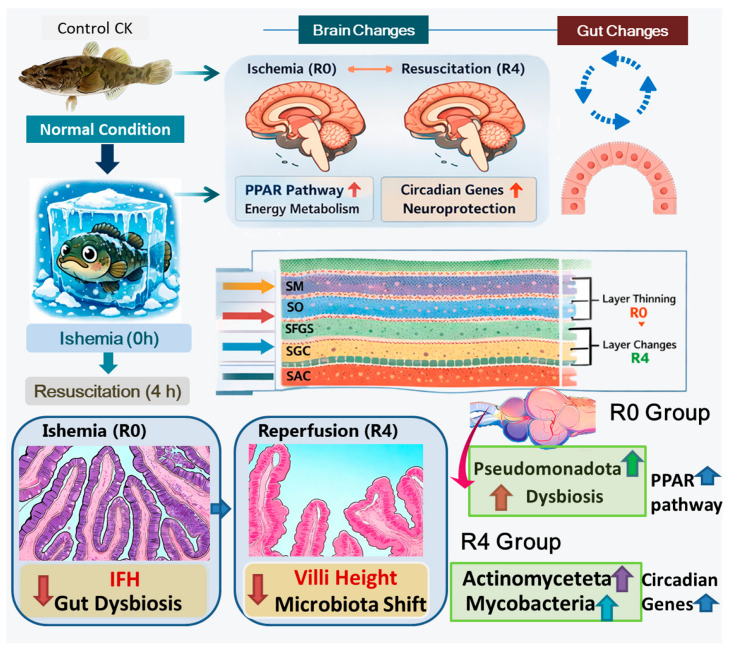
Integration analysis of brain transcriptome and gut microbiota.

The recovery of *P. glenii* from freezing is a complex physiological challenge that requires coordinated responses across multiple organ systems. Our findings suggest a dynamic, biphasic model in *P. glenii* in which the brain and gut, connected via the gut–brain axis, engage in a synchronized dialog to cope with the sequential stresses of ischemia (frozen) and reperfusion (thawing) (Figure 9). The present work integrated the observations of brain transcriptome remodeling and gut microbial shifts to propose a potential mechanism underlying extreme freeze tolerance in this species.

### The Ischemic Phase (R0): A Metabolic Dialog Centered on Energy Substrate Switching

Gerber et al. (2016) revealed the extreme adaptive strategies that life employs in response to harsh environments [45]. Insights from such adaptive mechanisms may help us understand why organisms fail to recover from freezing, a failure closely linked to apoptosis. Indeed, stressors during the recovery from freezing process, such as repeated recovery from freezing cycles and hypoxia/reoxygenation, serve as key triggers for the activation of apoptosis. A comprehensive understanding of these mechanisms is expected to facilitate the development of highly efficient cryoprotectants or technologies, ultimately reducing cell death in organs during freezing, transportation, and resuscitation. The cellular stress mechanisms involved in this process are closely associated with various human diseases, including ischemia–reperfusion injury [45,46,47]. One of the primary consequences of freezing is the induction of hypoxia and local ischemia: hypoxia is characterized by a deficiency of oxygen, and local ischemia is defined as impaired oxygen delivery resulting from interrupted blood flow [48]. Consequently, cryopreservation can be fundamentally regarded as an ischemic event [49], while the resuscitation corresponds to reperfusion. Reactive oxygen species (ROS) generated during reperfusion represent a major source of oxidative stress [50]. Several studies have confirmed an association between the gut microbiota of fish and stress [51,52,53]. However, the impact of recovery from freezing-induced dysbiosis on gut–brain communication remains unclear. Increasing evidence indicates that the gut microbiota regulates the host’s stress-related physiology and behavior through the gut–brain axis [54,55]. Therefore, understanding the coordinated response between the brain and gut is essential for elucidating the survival mechanisms of freeze-tolerant species. Numerous studies employing stress models have demonstrated that anxiety and depression-like behaviors, dysbiosis of gut microbiota, disruption of intestinal permeability, and alterations in the integrity of the blood–brain barrier (BBB) frequently occur concurrently [51,56]. Recent perspectives suggest that, in addition to being a critical factor influencing the neuro-immune–endocrine pathway, the gut microbiota may also directly regulate BBB integrity by modifying barrier permeability and tight junction function [57,58].

In the present study, we observed a significant enrichment of the PPAR signaling pathway in the brain tissue of *P. glenii* during the ischemic stage (R0 group). The peroxisome proliferator-activated receptor (PPAR) signaling pathway plays a central role in various physiological processes, including lipid metabolism, glucose homeostasis, cell differentiation, inflammatory response, and oxidative stress [59,60]. This pathway is vital for brain health, as it is expressed in various brain cells, such as astrocytes and neurons [61]. For instance, continuously upregulated genes in high-altitude *Bufo gargarizans* are enriched in fatty acid metabolism, vitamin absorption, cholesterol metabolism, and PPAR signaling, indicating their role in lipid metabolism and energy balance to aid hypoxia adaptation [62]. In the study of *Aplodinotus grunniens*, it was found that the PPAR pathway maintained the homeostasis of lipid and amino acid metabolism in freshwater drumfish under low temperatures [63]. Moreover, the primary physiological function of endocannabinoids may be to mediate the rapid regulation of synaptic input intensity by neurons [64]. Relevant studies indicate that in *Hypophthalmichthys molitrix* under hypoxia, semi-asphyxia, and asphyxia conditions, differentially expressed genes (DEGs) in the brain are significantly enriched in both the calcium signaling pathway and the retrograde endogenous cannabinoid signaling pathway [65]. Given that hypoxia also influences the freezing process in *P. glenii*, the findings of this study align with acute hypoxia research on *Pelteobagrus fulvidraco*, which shows that the retrograde endogenous cannabinoid signal is significantly enriched. This suggests that the acute hypoxia stage in fish can lead to neuronal dysfunction and alterations in synaptic plasticity [66]. In group R4, this study observed a significant enrichment of pathways associated with circadian rhythms. This finding is consistent with the cryopreservation study of *Rana sylvatica*, which indicated alterations in the expression of the core circadian rhythm proteins and their downstream target genes [67]. The brain tissues of *Pampus argenteus* were subjected to gradient cold exposure, and found that the miRNAs with differential expression under extreme cold stress were significantly enriched in the circadian rhythm pathway among their target genes [68]. This raises the possibility that the synchronization of circadian rhythms may play a role in regulating physiological adaptation and reprogramming gene expression during cryopreservation [67].

In the study of recovery from freezing in *P. glenii*, we observed that genes associated with the PPAR signaling pathway (PPARα, RXR, EHHADH, LPL, SLC27A, and PKC2) were significantly upregulated in the R0 group compared to the CK. In the context of our experimental conditions, this finding is of considerable significance. PPARα, the central nuclear receptor transcription factor in this pathway, primarily regulates the expression of genes involved in fatty acid oxidation [63] and plays a crucial role in modulating inflammatory responses and maintaining energy homeostasis [69,70]. In brain tissue, PPARα enhances mitochondrial function by upregulating mitochondrial enzymes and fatty acid transporter genes associated with lipid metabolism, thereby ensuring energy supply and mitigating nerve damage under ischemic conditions [71]. Consequently, the upregulation of PPARα during the R0 stage in *P. glenii* may provide essential energy support for the organism to counteract ischemic stress by promoting fatty acid oxidation in mitochondria and peroxisomes. Additionally, potential alterations in PPARα target genes (such as SCD1), which are often downregulated at low temperatures [72], may collaborate with these processes to maintain the dynamic balance of fatty acid composition during ischemia, regulate lipid metabolic homeostasis, and adapt to the demands of membrane fluidity in a hypothermic ischemic environment. The activation of the PPAR pathway in the R0 stage of *P. glenii* aligns with existing literature. Specifically, PPARs, as ligand-dependent transcription factors [73], can form heterodimers with RXR and bind to PPREs in the promoter region of target genes to regulate transcription [74]. PPAR ligands can reduce myocardial infarction area and serve as potential targets for anti-ischemic drugs [75]. Induced expression in the mouse brain is also beneficial for post-ischemic maintenance [74]. Drug studies, such as those involving Icariside II, demonstrate protective effects against cerebral ischemia/reperfusion injury by upregulating PPARα/γ and inhibiting NF-κB [76], further supporting the potential protective role of PPAR pathway upregulation in the R0 group of *P. glenii*. The observed stimulation of the PPAR-related axis may help to prevent oxidative damage and to maintain metabolic balance and cellular homeostasis in the brains of ectothermic zebrafish upon cold exposure [77]. Moreover, the PPAR signaling pathway regulates lipid breakdown and homeostasis in the brain by modulating genes such as EHHADH, which is crucial for maintaining nerve cells and blood–brain barrier (BBB) functions during the ischemic period [78]. The upregulation of the fatty acid transporter SLC27A observed in this study promotes the uptake of long-chain fatty acids [79]. This finding, combined with similar reports in *Litopenaeus vannamei* and *Cyprinus carpio* at low temperatures [80,81], suggests that *P. glenii* may adapt to energy requirements under ischemic stress by regulating the lipid metabolism rate overall during the R0 stage. Additionally, the mRNA levels of energy metabolism genes, such as the lipoprotein lipase gene LPL, were elevated in cold-resistant pufferfish [53]. LPL facilitates the release of fatty acids to maintain glucose homeostasis, thereby supporting thermogenesis [53,82]. The upregulation of the PPAR pathway in the R0 stage of the *P. glenii* may drive the expression of similar key energy metabolism genes (such as lpl), providing energy substrates for the organism. PCK2, a key enzyme in gluconeogenesis, serves not only as a component of the stress response and a newly discovered inhibitor of ferroptosis [83], but may also be an important factor mediating ischemic injury in astrocytes [84]. Its expression pattern in different recovery stages (R0 vs. R4) of *P. glenii* is worthy of attention and could be involved in the regulation of energy supply conversion and antioxidation/antiferroptosis. It is worth noting that during the resuscitation process of the *P. glenii*, when it entered the reperfusion stage (R4, resuscitation for 4 h), compared with the ischemic stage (R0), we observed a significant upregulation of circadian rhythm-related genes (PER1, PER2, PER3, RASD1, and MTNR1AA). This shift suggests the crucial role of the biological rhythm system in the later stage of recovery under our experimental conditions. The study of the wood frog (*Rana sylvatica*) provides a valuable reference: when it revives, the protein level of CRY-1 in the liver increases, but the nuclear localization and DNA binding activity of the core biological CLOCK complex BMAL-1/CLOCK are not significantly inhibited, and the expressions of PER-1 and PER-2 are disordered. This indicates that wood frogs may rely on non-classical mechanisms (such as post-translational modifications) to maintain the activity of biological clock proteins, thereby ensuring their metabolic regulation and anti-apoptotic functions during the resuscitation process. The upregulation of the circadian clock genes in the R4 stage of the *P. glenii* may reflect its attempt to reconstruct or adjust the circadian rhythm during the reperfusion stage to coordinate the metabolic recovery and cellular protection processes. Among them, Rasd1, as a newly discovered circadian clock regulatory gene [85], can affect neuronal excitability and response to stimuli by inhibiting the cAMP-PKA-CREB signaling pathway [86]. Its upregulation in the R4 stage suggests that it may play an important role in the recovery of neurological function in the later stage of resuscitation of *P. glenii*.

The dynamic transcriptional changes observed in the brain were accompanied by corresponding alterations in the intestine, which is consistent with a coordinated gut–brain response to freezing stress in this study. In the intestine, the morphological alterations are consistent with the established paradigm of ischemia–reperfusion injury. The significant reduction in intestinal fold height (IFH) observed specifically in the R4 (reperfusion) group compared to controls represents a critical finding. This observation aligns with the notion that intestinal structural damage induced by ischemia (R0 stage) may persist or even exacerbate during reperfusion. Dubois et al. (2025) reported that the reduction in intestinal fold height can be a delayed consequence of ischemic energy depletion and cellular damage [87]. Furthermore, Zhang et al. (2026) described severe pathology in murine models of intestinal ischemia–reperfusion, noting that “villi were damaged and shed,” which resulted in a loss of functional structure and a shortening of intestinal villi [88].

The gut microbiota, a key component of the gut–brain axis, also exhibited stage-specific shifts that paralleled the brain’s transcriptional reprogramming. At the level of gut microbiota, phylum-level analysis revealed that in the hypothermia treatment group, the abundance of *Bacillota* during the ischemic stage (R0 group) was significantly lower than that in the control group (CK) and the reperfusion stage (R4 group). Similarly, in the study of *Trachinotus ovatus*, the abundance of *Bacillota* increased under low-temperature stress [89]. Conversely, the abundance of *Pseudomonadota* in the R0 group was higher than that in both the CK and R4 groups. Feeding studies on mandarin fish (*Siniperca chuatsi*) demonstrated that the PPAR signaling pathway is a key regulator of lipid metabolism, with metabolic activity and *Pseudomonadota* abundance in the artificial feed group being significantly higher than in the natural feed group [90]. In the present study, the upregulation of the PPAR signaling pathway (related to lipid metabolism) in the R0 group was accompanied by a significant increase in the relative abundance of Pseudomonadota in the gut microbiota. This temporal coincidence suggests a potential functional linkage: the gut microbial community may contribute to the host’s metabolic adaptation during ischemia, possibly by modulating energy availability or producing signaling molecules that influence the brain’s PPAR-mediated response. Combined with previous studies indicating that Pseudomonadota abundance is associated with the regulation of host lipid metabolism, these results suggest a potential functional correlation between the PPAR pathway and Pseudomonadota in mediating freezing recovery, although direct regulatory mechanisms require further validation. The study on long kiss (*Leiocassis longirostris*) further confirmed the effect of temperature on the microbiota: under cold stress, the abundance of *Pseudomonadota* and *Bacillota* increased, while heat stress promoted the proliferation of *Fusobacteriota* and *Bacteroidota* [91].

It should be noted that the limitation of the present study was that the laboratory conditions did not simulate prolonged freezing in the field. Although our rapid freeze–thaw method is consistent with the biochemical work on *P. glenii* previously reported by Karanova et al. [92], that biochemical study did not examine neurogenomic or microbial responses. Similarly, field studies on Antarctic fishes involving freezing had longer freezing durations, but also did not include neurogenomic or microbial analyses [93,94]. Therefore, the present study can be considered a complement to those earlier works. Our experimental protocol was designed primarily to investigate the post-thaw recovery process; as such, the freezing phase itself and the mechanisms by which *P. glenii* survives prolonged freezing have not been fully elucidated. Future research should focus more directly on the freezing phase, employing longer and more ecologically relevant freezing durations, in order to more comprehensively clarify the freeze tolerance mechanisms of this species.

Under the specific freezing protocol applied in this study, the exceptional freeze tolerance of *P. glenii* appears to involve integrated and dynamic interplay between the brain and gut via the gut–brain axis, rather than being merely the sum of independent adaptations. We observe a biphasic, coordinated response: an initial ischemic phase marked by a PPAR-driven metabolic association between the brain and an altered gut microbiota, followed by a reperfusion phase characterized by circadian entrainment in the brain and concurrent structural and microbial remodeling in the gut. These findings contribute to a systems-level understanding of freeze tolerance in this vertebrate species, and suggest the brain–gut axis as a potential regulatory platform. Within the limitations of our correlational design, these results offer new insights into the molecular and ecological basis of surviving ischemia–reperfusion events in *P. glenii*, and point to hypotheses that can be tested in future studies with causal interventions [94].

## 5. Conclusions

This study provides comprehensive evidence that the exceptional freezing tolerance of *Perccottus glenii* relies on a finely orchestrated interplay between neural regulation and gut microbial ecology. The brain responds to freezing-induced ischemia through rapid upregulation of the PPAR signaling pathway to maintain energy homeostasis and mitigate neuronal damage, followed by a later activation of circadian rhythm genes that may facilitate metabolic recovery during reperfusion. Parallel restructuring of the gut microbiota community, particularly the fluctuations in Bacillota and Pseudomonadota abundances, further underscores the ecological dimension of freezing recovery. These findings significantly advance our understanding of adaptive strategies to extreme cold, suggesting the brain–gut axis as a potential key regulator of freeze tolerance in *P. glenii* and offering new insights into the potential molecular basis of ischemia–reperfusion recovery in this species, with broader biological and biomedical implications.

## Figures and Tables

**Table 1:** qPCR primers for detecting brain transcriptomic genes.

NAME	Sequence (5′-3′)	Product Length (bp)	Amplification Efficiency (%)
PPARA	ACAAGTGCCAGTTCTGCCGATTC	144	107.9
	TGGGGTTTGGTTCCTCCTTCTCC		
LPL	AGAGGTGGAGCGACGCAGATAC	137	104.5
	TCTCAACCTCCAGCCAGTGTCTC		
PCK2	TCCCACCTGCCCGACACAAG	130	108.7
	CTGCCAGCCAGCCTTCATCTTTAG		
EHHADH	GTGCCGATGATCCATGCCATAGAG	129	108.5
	TCTGGCTTTGGAGTGTGCGATTC		
SLC23A2	ATCACAACATCCGCACCAAGTCTC	101	116.8
	CACGTCCTCCACAGCAGTTCTTAG		
RXRBA	GATACGGGACAGTGGAGTTCAAGC	141	104.5
	GTCGTGGCAGCAGCAGTGAC		
RXRAA	TCGTTCTCGCACCGTTCAATATCG	138	110.6
	CATCTTCGACACCAGCTCCGTTAG		

**Table 2:** Summary of transcriptome assembly.

Samples	Raw Data	Clean Data	Ratio	Q20 (%)	Q30 (%)	GC (%)
CK-1	41,108,032	40,768,848	99.17%	96.78	91.48	43.94
CK-2	37,626,788	37,304,266	99.14%	96.73	91.35	44.15
CK-3	41,585,312	41,220,966	99.12%	96.54	90.46	43.19
R0-1	38,873,032	38,829,616	99.89%	98.93	96.99	46.76
R0-2	47,063,206	47,010,156	99.89%	99.05	97.37	45.73
R0-3	46,746,908	46,692,196	99.88%	99.03	97.28	47.09
R4-1	43,580,364	43,247,602	99.24%	97.11	92.09	44.42
R4-2	42,495,622	42,082,112	99.03%	96.69	91.21	44.58
R4-3	40,217,382	39,862,084	99.12%	96.66	90.77	44.54

## Data Availability

Data involved in this present study were deposited in the Sequence Read Archive (SRA) (https://www-ncbi-nlm-nih-gov.ezproxy.its.uu.se/sra, accessed on 18 January 2026). The accession number of our submission is SUB15647090.

## Supplementary Materials

The following supporting information can be downloaded at: https://www.mdpi.com/article/10.3390/ani16091338/s1, Table S1: This table presents the differential expression analysis results of various genes across three sample groups (CK-R0, CK-R4, R0-R4), including the following core information: log2(fc), PValue and description.
