# Supplementary material for: Integrated Gut–Brain Axis Response to Freezing and Recovery in Freeze-Tolerant Fish, Perccottus glenii
Source: Animals (Basel). 2026 Apr 27;16(9):1338. doi: 10.3390/ani16091338 (PMC13163002; doi:10.3390/ani16091338)
Supplement: Supplementary file 1 [file animals-16-01338-s001.zip › animals-4270027-supplementary.pdf]

Table S1. This table presents the differential expression analysis results of various genes across three sample groups (CK-R0, CK-R4, R0-R4), including the following core information: log2(fc), PValue and description.

id	Symbol	CK-R0 log2(fc)	PValue	CK-R4 log2(fc)	PValue	R0-R4 log2(fc)	PValue	Description
GCA_024416835.2002089	PPARA	1.323505	2.97E-11	0.422438	0.011036	-0.90107	1.81E-05	peroxisome proliferator-activated receptor alpha a isoform X1
GCA_024416835.2020681	PRKG1	-3.24025	4.69E-15	0.736325	0.083262	3.078671	1.31E-16	cGMP-dependent protein kinase 1
GCA_024416835.2007729	PRKG1	-1.506	0.283104	-0.16158	0.657398	2.24233	0.015201	cGMP-dependent protein kinase 1 isoform X2
GCA_024416835.2014114	LPL	1.264358	3.87E-10	0.010646	0.146362	-1.25371	1.06E-07	lipoprotein lipase
GCA_024416835.2013390	PCK2	2.53179	0.36E-4	-1.31467	0.634448	-3.11304	3.28E-05	phosphoenolpyruvate carboxykinase [GTP], mitochondrial
GCA_024416835.2014855	EHHADH	1.548035	0.001626	0.096875	0.621513	-1.45116	0.00286	peroxisomal bifunctional enzyme
GCA_024416835.2003714	SLC27A2	3.161734	0.044865	0.755228	0.753564	-2.40651	0.096382	long-chain fatty acid transport protein 2 isoform X2
GCA_024416835.2016591	RXRBA	1.567181	3.92E-06	0.250475	0.323521	-1.31671	0.000299	retinoic acid receptor RXR-beta-A isoform X3
GCA_024416835.2002878	RXRAA	1.718892	0.004188	-0.78909	0.60749	-2.50798	0.001201	retinoic acid receptor RXR-alpha isoform X4
GCA_024416835.2000474	PER1	-1.48752	4.99E-08	-0.20442	0.677639	1.283097	4.72E-08	period circadian protein homolog 1b isoform X2
GCA_024416835.2012315	PER2	-1.70932	0.02537	-0.09262	0.534619	1.765409	0.002315	period circadian protein homolog 2
GCA_024416835.2001150	PER3	-1.4579	0.00827	0.36057	0.01950	1.81847	4.26E-06	period circadian protein homolog 3 isoform X1

					5	1		
GCA_024416835.2020222	RASD1	-2.98141	3.45E-11	-0.26034	0.95257	3.33272	2.08E-12	dexamethasone-induced Ras-related protein 1
GCA_024416835.2005540	RYR1	-2.37547	4.46E-06	0.017728	0.063768	3.061593	1.3E-14	ryanodine receptor 1 isoform X13
GCA_024416835.2004188	MTNR1AA	-3.97787	0.091498	1.446652	0.039173	5.424525	0.001157	melatonin receptor type 1A
